# Early Spontaneous Abdominal Bleeding is associated with Poor Outcome in Moderate to Severe Acute Pancreatitis Patients: A Propensity Matched Study

**DOI:** 10.1038/srep42607

**Published:** 2017-02-22

**Authors:** Yizhe Chen, Jing Zhou, Gang Li, Zhihui Tong, Jie Dong, Yiyuan Pan, Lu Ke, Weiqin Li, Jieshou Li

**Affiliations:** 1 Department of General Surgery, Jinling Hospital, Medical School of Nanjing University, No. 305 Zhongshan East Road, Nanjing, China

## Abstract

Abdominal bleeding is a lethal complication in acute pancreatitis (AP) and it is commonly described as a late event. However, spontaneous intra-abdominal bleeding could occur very early but no study focusing on this phenomenon was published yet. In this study, 1137 AP patients were retrospectively screened and 24 subjects suffering early spontaneous bleeding (ESB) were selected. Meanwhile, a 1:1 well-balanced cohort of non-bleeding patients was generated by propensity score match. The clinical characteristics of these patients were compared and a multiple regression analysis was performed to assess the risk factors for ESB. Besides, patients with massive post-intervention bleeding (PIB) were collected for additional comparison. ESB patients suffered significantly worse outcome than the matched cohort evidenced by dramatically higher mortality than the non-bleeding patients and even the PIB group (54.2% versus 20.8%, *P* = 0.017; 54.2% versus 31.0%, *P* = 0.049). The regression analysis demonstrated computer tomography severity index (CTSI; OR, 3.34; 95% CI, 1.995–5.59, *P* < 0.001) and creatinine (OR, 1.008; 95% CI, 1.004–1.012, *P* < 0.001) were associated with the occurrence of ESB. In conclusion, ESB is a rare but dangerous complication of moderate-to-severe AP and may result in high mortality. CTSI and creatinine are independent risk factors for the development of ESB.

Acute pancreatitis (AP) is a sudden inflammatory disorder of the pancreas, among which, quite a number of cases are self-limited and resolve without severe complications. However, severe acute pancreatitis (SAP) develops in 15–20% patients and is associated with a mortality between 20% to 30%^1^. Mortality in SAP occurs either early, owing to multiple organ dysfunction syndrome (MODS) or late, due to septic shock and uncontrolled major bleeding.

Intra-abdominal bleeding is a fatal complication of AP, which was considered as the primary cause for >50% death cases of AP^2^,^3^. Up till now, it is commonly considered that intra-abdominal bleeding usually develop late in the clinical course^2^,^4^,^5^. According to the study conducted by Balthazar *et al*.^5^, bleeding complications were detected from 2 months to 8 years after one or several episodes of pancreatitis. Flati^2^ and his colleagues summarized massive intra-abdominal bleeding in AP patients and found more than 60% bleeding events occur as a complication of necrosis. It has long been thought that the local inflammation and necrosis in AP would increase the risk of vessel wall injury and finally lead to the bleeding. That is easy to explain why intra-abdominal bleeding usually occur late in the disease course.

However, we noticed that spontaneous intra-abdominal bleeding could occur infrequently at relatively early phase and there was no previous study describing this phenomenon probably due to its limited incidence. Therefore in the present study, we aimed to explore the clinical significance of early spontaneous intra-abdominal bleeding (ESB) in patients with moderate to severe acute pancreatitis using propensity score matched analysis.

## Results

### Study Population

**Figure 1:**
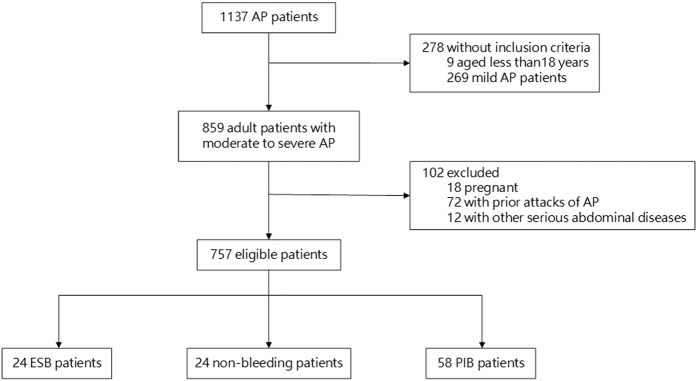
Study flowchart.

As shown in the Fig. 1, among all 1137 AP patients, 757 eligible patients who met the inclusion and exclusion criteria were screened. Of whom, 24 ESB patients were selected for final analysis. Each ESB patient was exactly and propensity score matched 1:1 with patients without intra-abdominal bleeding (non-bleeding group). In addition, 58 patients who suffered massive post-intervention bleeding (PIB) were enrolled into the PIB group for an additional comparison.

### Variables between ESB patients and non-bleeding patients

The baseline characteristics including age, gender, body mass index (BMI), time interval from AP onset to admission, etiology and co-morbidities between ESB patients and non-bleeding patients were displayed in Table 1 and no significant difference was found. Table 2 showed the admission status (vital signs, laboratory data, and severity scores, etc.) of ESB patients and non-bleeding patients. Despite the propensity score match we made, significant differences could still be found in the following aspects: blood urea nitrogen (10.7[4.8–20.6] vs. 5.6[2.8–9.5], *P* = 0.008), creatinine (207.5[58.8–339.5] vs. 58[38–114], *P* = 0.007) and computer tomography severity index (CTSI; 10[10–10] vs. 8[6–10], *P* = 0.005).

The disease course and prognosis of ESB patients were largely different from the non-bleeding patients. As shown in the Table 3, ESB patients had a higher risk of developing organ failures including shock, acute kidney injury (AKI), acute liver injury (ALI) and multiple organ dysfunction syndrome (MODS) than non-bleeding patients. Moreover, ESB patients suffered more invasive treatment measures including continuous renal replacement therapy (CRRT), percutaneous catheter drainage (PCD), negative pressure irrigation (NPI) and digital subtraction angiography (DSA). In addition, the proportion of severe acute pancreatitis in ESB patients was significantly higher than non-bleeding patients (22, 91.7% vs. 11, 45.8%, *P* = 0.001), so were the mortality (13, 54.2% vs. 5, 20.8%, *P* = 0.017), intensive care unit (ICU) duration and cost.

### Risk factors predicting ESB

A multivariable regression model including 16 indices (age, gender, etiology of AP, acute physiology and chronic health evaluation II score, etc.) was performed to evaluate risk factors of ESB. Univariate logistic regression analysis (Table 4) revealed significant correlations between ESB and prothrombin time (PT; *P* = 0.027), creatinine (*P* = 0.011) and CTSI (*P* < 0.001). Taking the three significant variables together into the multiple logistic regression model, CTSI (OR, 3.34; 95% CI, 1.995–5.59, *P* < 0.001) and creatinine (OR, 1.008; 95% CI, 1.004–1.012, *P* < 0.001) were proved to be independent risk factors for ESB (shown in Table 5).

### Variables between ESB patients and post-intervention patients

**Figure 2:**
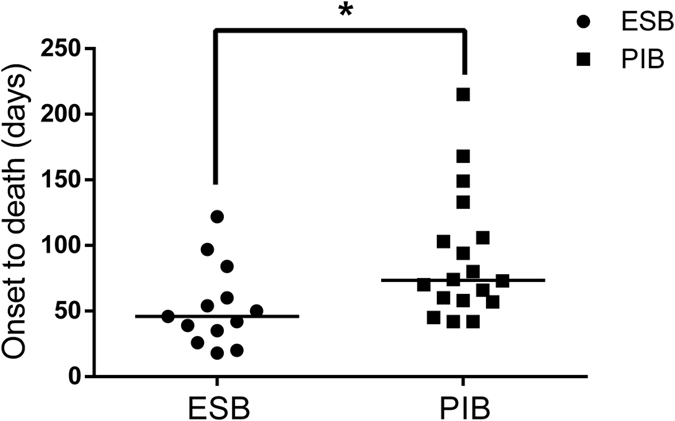
Distribution of survival time between ESB group and PIB group (Mann–Whitney U-test). The lines in the figure refer to the median survival time of patients (ESB group: 46[30.5–72] days vs PIB group: 73.5[57.75–112.75] days, *P* = 0.010).

As shown in the Table 1, there was no significant difference regarding the baseline characteristics between ESB and PIB groups. Besides, Table 6 showed the admission status and prognosis of the two groups, among which, significant difference could be detected in the following aspects: creatinine, acute physiology and chronic health evaluation (APACHE) II score, time interval from AP onset to bleeding, infected pancreatic necrosis (IPN), acute liver injury (ALI) and mortality, indicting a more severe clinical course of ESB patients even compared with PIB patients. Unexpectedly, the occurrence of ESB was associated with an even lower IPN rate. Furthermore, as Fig. 2 showed, the time interval from disease onset to death of ESB patients was significantly shorter than PIB patients (46[30.5–72] days versus 73.5[57.75–112.75] days, *P* = 0.010). Additionally, there were 17 patients(26 times) in the ESB group and 50 patients(70 times) in the PIB group received digital subtraction angiography (DSA) examination, respectively, among which, positive findings were found in 15 (14 artery and 1 vein) cases and 35 (33 artery and 2 vein) cases. Transcatheter arterial embolization (TAE) was successfully performed for 13 times in 9 of the 24 ESB patients and 27 times in 22 of the 58 PIB patients.

## Discussion

In this study, early spontaneous intra-abdominal bleeding developed in 24 of all 757 moderate to severe AP patients (3.2%). The mortality of ESB patients was up to 54%, which was extremely high considering the mortality of AP patients in our department was only 6~8% during the study period. With almost the same baseline characteristics and admission status, ESB patients suffered much more severe disease compared with not only non-bleeding patients but also PIB patients, suggesting ESB is a rare but lethal complication of AP.

The multivariable regression model demonstrated that a high CTSI (more pancreatic necrosis) and a high creatinine level (worse renal function) may lead to the development of ESB. As far as we know, the erosion and rupture of blood vessel and pseudoaneurysm which resulted from severe pancreatic necrosis, abscesses, and pseudocysts, local inflammation and aggressive surgical intervention were the leading causes of intra-abdominal bleeding in AP patients^2^,^4^,^6^,^7^,^8^. According to the previous study, local infected fluid collections (necrosis, pseudocyst, and abscess) were the most common risk factors for hemorrhage in AP which account for more than 60% cases^2^. In this regard, it is easy to explain why intra-abdominal bleeding is more likely to happen in patients with more pancreatic necrosis, namely, a high CTSI. Besides, bleeding event was also considered as a major complication of renal failure, especially in the pre-dialysis era^9^,^10^. Although, the underling mechanism of hemorrhage in patients with kidney injury is still unclear, the need for anticoagulation like heparin during the renal replacement therapy and effects related to uraemic thrombopathy may be two primary causes^11^,^12^,^13^,^14^. Our previous work also showed AKI was an independent risk factor for intra-abdominal bleeding in AP patients^15^. However, the cause-and-effect relationship between renal failure and bleeding event remains unclear, intra-abdominal bleeding may also severely influence the renal function due to the accompanying volume deficiency, intra-abdominal hypertension, and secondary infection, etc.

As mentioned before, the development of intra-abdominal bleeding in AP patients was considered as a progressive process caused by persistent accumulation of vessel injuries. Thus, intra-abdominal bleeding tends to occur late in the course of AP, which has already been described in a series of previous studies^2^,^4^,^5^. However, in this study, the median time interval from AP onset to bleeding event of ESB patients was only 17.5 (10–20) days, dramatically shorter than PIB patients (51[26.75–70.75] days). Besides, the statistical results indicted ESB patients suffered significantly worse prognosis than PIB patients, except for a lower IPN rate which may be attributed to the shorter interval from disease onset to death in some ESB patients. The exact cause of ESB remains unknown, but activated pancreatic enzymes might play a role in the development of ESB. It is known that extravasation of exocrine enzyme-rich fluids within areas of parenchymal necrosis may further aggravates the necrotizing process and increases the risk of damaging the walls of vessels^16^. Considering the more pancreatic necrosis ESB patients suffered, they should had a high local enzyme level, which may contribute substantially to the occurrence of ESB. Taking together, the underlying mechanism of ESB may be different from PIB and further studies are required.

Certain limitations of this study need to be addressed. Due to the small number of study patients and limited incidence of ESB, the statistical power of the analyses could be relatively weak and some of the findings may be not significant in a larger sample. Moreover, as a retrospective study, selection bias can hardly be avoided especially between the ESB group and PIB group which we could not conduct a propensity score match because of limited sample size.

## Conclusion

Early spontaneous bleeding is a rare but potentially lethal complication of AP which occurs in approximately 3% of moderate to severe AP patients, and is associated with a high mortality of 54%. CTSI and creatinine are independent risk factors for the development of ESB in AP patients.

## Methods

This retrospective propensity matched cohort study was conducted at the Department of General Surgery in Jinling Hospital between January 2013 and December 2015. Jinling Hospital is a teaching hospital affiliated to the Nanjing University, and serves patients from various regions all over China. As a retrospective study, no ethics approval is required in our institute. To retrieve data from the electric database, we ask for an approval from the Acute Pancreatitis Database Management Committee. All the analyses were performed in compliance with the committee’s regulation. Informed consent regarding data storage and publication was obtained from each patients who were recorded in the database during their hospitalization.

### Definitions

Different diagnostic approaches of spontaneous bleeding were described previously^3^,^17^,^18^. In our study, early spontaneous bleeding (ESB) was defined when the bleeding could be detected on contrast-enhanced computed tomography (CECT) within 30 days from initial presentation without prior minimally-invasive or operative intervention. Considering the bleeding risk resulted from surgical interventions^2^,^7^,^8^,^19^,^20^, we had excluded patients with prior intervention to reduce the bias. CT scans for patients were reviewed separately by two radiologists with rich experience in abdominal imaging. Surgical interventions including percutaneous catheter drainage (PCD), negative pressure irrigation (NPI), endoscopic necrosectomy (EN), and laparotomy were considered when patients were diagnosed with infected pancreatic necrosis, digestive tract fistula, persistent abdominal compartment syndrome (ACS), and/or uncontrolled hemorrhage and so on. Besides, patients who suffered massive intra-abdominal bleeding after any abovementioned surgical intervention were collected as the post intervention bleeding (PIB) group for an additional comparison. The definition of massive bleeding was a decrease of hemoglobin concentration >2 g/dL and/or significant hemodynamic deterioration caused by intra-abdominal bleeding^2^,^15^. Organ failures including shock, acute respiratory distress syndrome (ARDS), acute kidney injury (AKI) and acute liver injury (ALI) were assessed. Shock was defined as systolic blood pressure <90 mmHg or need for inotropic agent, respiratory failure was defined as PaO2/FiO2 ≤ 300 mmHg, renal failure was defined as serum creatinine ≥176umol/L (2.0 mg/dL) and the criteria for hepatic failure was defined as a score of ≥2 using the Marshall scoring system^21^. The diagnoses of local and systemic complications were made based on the revised Atlanta classification of acute pancreatitis^22^. According to the guidelines of World Society of Abdominal Compartment Syndrome^23^, abdominal compartment syndrome (ACS) was defined as a sustained intra-abdominal pressure more than 20 mmHg accompanied with new-onset organ dysfunction/failure. CTSI was evaluated according to the contrast-enhanced computed tomography using the Balthazar’s CT score^24^.

### Patients

We screened all consecutive adult patients (age ≥ 18 years) who were diagnosed with moderate to severe AP in our center between January 2013 and December 2015. Patients with mild AP were not included because they rarely suffered intra-abdominal bleeding thus may bring in additional selection bias. Diagnostic criteria for moderate to severe AP were defined according to the revised Atlanta criteria^22^. Exclusion criteria included pregnancy, prior attacks of acute pancreatitis, and combining with other serious abdominal diseases. Finally, 757 eligible patients were enrolled for further analysis.

Early spontaneous bleeding could be defined in 24 patients (ESB group). Every case was 1:1 propensity score matched with another patient without intra-abdominal bleeding (non-bleeding group). Matching was based on the admission APACHE II score, demographics (age, gender, and body mass index), time interval from AP onset to admission, and etiology (biliary, hypertriglyceridemia, alcoholic, and other). The propensity score match could effectively decrease bias and improve control for confounding variables^25^. To compare with patients who suffered “classic” AP-related bleeding and avoid potential overlap, 58 patients who suffered massive intra-abdominal bleeding after surgical intervention (PIB group) was additionally studied to compare with the ESB group in terms of clinical prognosis. All patients received the same standard^26^,^27^ treatment including fluid therapy, pain control, nutritional support, antibiotics and so on. In patients with massive intra-abdominal bleeding, resuscitation was applied at the first time, digital subtraction angiography (DSA) and subsequent transcatheter arterial embolization (TAE) were attempted consecutively if possible. Laparotomy was only performed in patients who cannot be controlled with non-operative measures. DSA was also applied in some non-bleeding patients.

### Data Collection

The data of patients was obtained from our AP database which contains information of more than two thousand AP patients. Collected variables included demographics, medical history, etiology, co-morbidities, vital signs, laboratory tests (blood routine examination, blood biochemistry, coagulation routine, etc.), imaging data, systemic and local complications, special treatments, and outcomes (mortality, hospital and intensive care unit durations, cost, etc.). The Acute Physiology and Chronic Health Evaluation (APACHE) II score and Sequential Organ Failure Assessment (SOFA) score were also acquired from the database.

### Statistical Analysis

All analyses were performed using SPSS 22.0 for windows (IBM Analytics, Armonk, NY). Data are expressed as median (interquartile range) for continuous variables and frequencies (proportions) for categorical variables. Mann–Whitney U-test and Pearson test was used as the circumstances required. To identify the risk factors for ESB, several series of univariate logistics regression analyses using 16 indices were performed. Variables that showed statistical significance were tested in further multiple logistic regression analyses. Odds ratio was expressed with 95% confidence interval (CI). All statistical tests were two-tailed, and the statistical significance was considered as *P* < 0.05.

## Additional Information

**How to cite this article**: Chen, Y. *et al*. Early Spontaneous Abdominal Bleeding is associated with Poor Outcome in Moderate to Severe Acute Pancreatitis Patients: A Propensity Matched Study. *Sci. Rep.* **7**, 42607; doi: 10.1038/srep42607 (2017).

**Publisher's note:** Springer Nature remains neutral with regard to jurisdictional claims in published maps and institutional affiliations.

## Figures and Tables

**Table 1:** Baseline characteristics of ESB, Non-bleeding, and PIB Patients.

Variable	ESB Group (n = 24)	Non-bleeding Group (n = 24)	*P* Value*	PIB Group (n = 58)	*P* Value†
Age, years	43 (36–47.25)	45.5 (34.75–49.5)	NS (0.570)	46 (39–51)	NS (0.155)
Gender, M/F	19/5	17/7	NS (0.505)	48/10	NS (0.702)
BMI, kg/m^2^	24.8 (22.2–28.5)	25.5 (21.17–27.75)	NS (0.836)	25.25 (23.08–27.59)	NS (0.967)
Onset to admission, days	18.5 (5.75–26.5)	10 (6–41.25)	NS (0.844)	23 (9.5–40)	NS (0.089)
Etiology, no. (%)			NS (0.499)		NS (0.99)
Biliary	13 (54.2)	16 (66.7)		32 (55.2)	
HTG	9 (37.5)	8 (33.3)		20 (34.5)	
Alcoholic	1 (4.2)	0 (0)		3 (5.2)	
Other	1 (4.2)	0 (0)		3 (5.2)	
Co-morbidities, no. (%)					
Smoking	10 (41.7)	8 (33.3)	NS (0.551)	29 (50.0)	NS (0.492)
Alcohol use	12 (50)	9 (37.5)	NS (0.383)	24 (41.4)	NS (0.474)
Hypertension	7 (29.2)	11 (45.8)	NS (0.233)	19 (32.8)	NS (0.750)
Diabetes mellitus	5 (20.8)	7 (29.2)	NS (0.505)	9 (15.5)	NS (0.560)
Coronary heart disease	0 (0)	2 (8.3)	NS (0.149)	4 (6.9)	NS (0.187)
Fatty liver	3 (12.5)	5 (20.8)	NS (0.439)	12 (20.7)	NS (0.383)
Chronic respiratory disease	2 (8.3)	3 (12.5)	NS (0.637)	8 (13.8)	NS (0.492)
Chronic renal disease	0 (0)	1 (4.2)	NS (0.312)	1 (1.7)	NS (0.517)

*BMI* body mass index, *HTG* hypertriglyceridemia. *ESB Group versus Non-bleeding Group, †ESB Group versus PIB Group. Data are expressed as median (interquartile range) for continuous variables and frequencies (proportions) for categorical variables, as appropriate. Mann–Whitney U-test and Pearson test was used as the circumstances required.

**Table 2:** Comparison of ESB patients and Non-bleeding patients on admission.

Variable	ESB Group (n = 24)	Non-bleeding Group (n = 24)	*P* Value
Temperature, °C	37.85 (36.53–38.58)	37.5 (37.08–38.2)	NS (0.702)
MAP, mmHg	94.33 (73.33–108.67)	93.33 (87.92–103.83)	NS (0.885)
Heart rate	113.5 (100.5–131.5)	114 (91.25–127.75)	NS (0.680)
Respiratory rate	26 (22.25–34.25)	24 (18.25–34.5)	NS (0.337)
IAP, mmHg	11.7 (8.7–14.65)	9.15 (7.45–12.0)	NS (0.066)
Hemoglobin, g/L	81.5 (73.25–99)	91.5 (78–99.0)	NS (0.312)
Platelet count, x10^9^ cells/L	188 (135.75–299.5)	203 (121.75–278)	NS (0.975)
PT, s	13.65 (12.8–14.75)	14.45 (13.03–16.6)	NS (0.208)
APTT, s	36.6 (31.83–42.33)	36.75 (28.6–43.53)	NS (0.902)
INR	1.2 (1.11–1.4)	1.23 (1.16–1.29)	NS (0.628)
C-reactive protein, mg/L	125.9 (89.9–182.1)	117.7 (80.8–183.15)	NS (0.585)
Total bilirubin, umol/L	26.3 (15.6–83.0)	21.8 (10.78–52.98)	NS (0.364)
Unconjugated bilirubin, umol/L	10.9 (5.0–34.5)	7.0 (2.93–13.9)	NS (0.197)
ALT, U/L	24.5 (12.25–48.25)	32.5 (25.25–60.5)	NS (0.173)
AST, U/L	29 (22.5–88.5)	37 (25.25–67)	NS (0.585)
BUN, mmol/L	10.7 (4.8–20.6)	5.6 (2.8–9.5)	0.008
Creatinine, umol/L	207.5 (58.8–339.5)	58 (38–114)	0.007
CTSI	10 (10–10)	8 (6–10)	0.005
APACHE II score	15.5 (9–22.3)	15.5 (10.25–22.75)	NS (0.885)
SOFA score	6 (2.25–8.75)	3 (2–6)	NS (0.076)

*MAP* mean arterial pressure, *IAP* intra-abdominal pressure, *PT* prothrombin time, *APTT* activated partial thromboplastin time, *INR* international normalized ratio, *ALT* alanine transaminase, *AST* aspartate transaminase, *BUN* blood urea nitrogen, *CTSI* computer tomography severity index, *APACHE* acute physiology and chronic health evaluation, *SOFA* sequential organ failure assessment. Data are expressed as median (interquartile range) for continuous variables and frequencies (proportions) for categorical variables, as appropriate. Mann–Whitney U-test and Pearson test was used as the circumstances required.

**Table 3:** Clinical course and outcome between ESB patients and Non-bleeding patients.

Variable	ESB Group (n = 24)	Non-bleeding Group (n = 24)	*P* Value
IPN, no. (%)	19 (79.2)	15 (62.5)	NS (0.204)
Digestive tract fistula, no. (%)	10 (41.7)	6 (25.0)	NS (0.221)
SVT, no. (%)	5 (20.8)	5 (20.8)	NS (1)
ACS, no. (%)	7 (29.2)	0 (0)	0.004
Organ failure			
Shock, no. (%)	16 (66.7)	8 (33.3)	0.021
ARDS, no. (%)	19 (79.2)	15 (62.5)	NS (0.204)
AKI, no. (%)	17 (70.8)	10 (41.6)	0.042
ALI, no. (%)	13 (54.2)	6 (25.0)	0.039
MODS, no. (%)	18 (75.0)	8 (33.3)	0.004
Management			
CRRT, no. (%)	16 (66.7)	8 (33.3)	0.021
PCD, no. (%)	20 (83.3)	13 (54.2)	0.029
NPI, no. (%)	18 (75.0)	8 (33.3)	0.004
ED, no. (%)	11 (45.8)	8 (33.3)	NS (0.376)
DSA, no. (%)	17 (70.8)	5 (20.8)	0.001
Laparotomy, no. (%)	12 (50.0)	6 (25.0)	NS (0.074)
Revised Atlanta classification			0.001
Mild, no. (%)	0 (0)	0 (0)	
Moderate severe, no. (%)	2 (8.3)	13 (54.2)	
Severe, no. (%)	22 (91.7)	11 (45.8)	
Mortality, no. (%)	13 (54.2)	5 (20.8)	0.017
Hospital duration, days	35 (19.5–83.75)	13.5 (8–64.5)	NS (0.060)
ICU duration, days	24.5 (12.75–46.25)	9 (4.25–33.75)	0.042
Cost, thousand CNY	293 (183–459)	120 (49–449)	0.026

*IPN* infected pancreatic necrosis, *SVT* splanchnic venous thrombosis, *ACS* abdominal compartment syndrome, *ARDS* acute respiratory distress syndrome, *AKI* acute kidney injury, *ALI* acute liver injury, *MODS* multiple organ dysfunction syndrome, *CRRT* continuous renal replacement therapy, *PCD* percutaneous catheter drainage, *NPI* negative pressure irrigation, *ED* endoscopic necrosectomy, *DSA* digital subtraction angiography, *ICU* intensive care unit. Data are expressed as median (interquartile range) for continuous variables and frequencies (proportions) for categorical variables, as appropriate. Mann–Whitney U-test and Pearson test was used as the circumstances required.

**Table 4:** Univariate logistic regression analysis of ESB.

Variable	Odds Ratio	95% CI		*P* Value
		Lower	Upper	
Age	0.955	0.896	1.018	0.155
Gender	1.105	0.141	8.679	0.925
Etiology	1.136	0.399	3.234	0.812
Smoking	0.978	0.162	5.909	0.981
Alcohol use	0.169	0.023	1.236	0.080
Platelet count	1.002	0.995	1.009	0.611
PT	0.444	0.216	0.913	0.027
APTT	0.997	0.954	1.042	0.889
INR	1497.253	0.385	5822920.583	0.083
C-reactive protein	0.991	0.979	1.004	0.173
Total bilirubin	0.996	0.982	1.010	0.585
Unconjugated bilirubin	1.050	0.982	1.123	0.152
BUN	0.962	0.772	1.198	0.730
Creatinine	1.018	1.004	1.032	0.011
CTSI	5.764	2.378	13.971	<0.001
APACHE II score	0.906	0.793	1.036	0.148

*PT* prothrombin time, *APTT* activated partial thromboplastin time, *INR* international normalized ratio, BUN blood urea nitrogen, *CTSI* computer tomography severity index, APACHE acute physiology and chronic health evaluation.

**Table 5:** Independent prognostic factors in a multivariate logistic regression analysis of ESB.

Variable	Odds Ratio	95% CI		*P* Value
		Lower	Upper	
CTSI	3.340	1.995	5.590	<0.001
Creatinine	1.008	1.004	1.012	<0.001

*CTSI* computer tomography severity index.

**Table 6:** Admission status, clinical course and outcome between ESB patients and PIB patients.

Variable	ESB Group (n = 24)	PIB Group (n = 58)	*P* Value
Hemoglobin, g/L	81.5 (73.25–99)	91 (79.75–101.5)	NS (0.142)
Platelet count, ×10^9^ cells/L	188 (135.75–299.5)	186 (106.75–284)	NS (0.795)
PT, s	13.65 (12.8–14.75)	13.45 (12.15–14.8)	NS (0.71)
APTT, s	36.6 (31.83–42.33)	33.5 (27.95–40.35)	NS (0.154)
INR	1.2 (1.11–1.4)	1.17 (1.055–1.2725)	NS (0.177)
C-reactive protein, mg/L	125.9 (89.9–182.1)	131.45 (96.675–191)	NS (0.744)
Total bilirubin, umol/L	26.3 (15.6–83)	24.15 (13.5–55.25)	NS (0.488)
Unconjugated bilirubin, umol/L	10.9 (5.0–34.5)	6.55 (3.55–13.075)	NS (0.06)
ALT, U/L	24.5 (12.25–48.25)	32 (21–56)	NS (0.243)
AST, U/L	29 (22.5–88.5)	34.5 (24–54.25)	NS (0.919)
BUN, mmol/L	10.7 (4.8–20.6)	9.1 (5.35–14.725)	NS (0.415)
Creatinine, umol/L	207.5 (58.8–339.5)	81.5 (49.5–188.5)	0.037
APACHE II score	15.5 (9–22.3)	11.5 (8.75–15)	0.032
Onset to bleeding, days	17.5 (10–20)	51 (26.75–70.75)	<0.001
IPN, no. (%)	19 (79.2)	56 (96.6)	0.010
Digestive tract fistula, no. (%)	10 (41.7)	28 (48.3)	NS (0.585)
SVT, no. (%)	5 (20.8)	13 (22.4)	NS (0.875)
ACS, no. (%)	7 (29.2)	10 (17.2)	NS (0.225)
Organ failure			
Shock, no. (%)	16 (66.7)	37 (63.8)	NS (0.804)
ARDS, no. (%)	19 (79.2)	39 (67.2)	NS (0.280)
AKI, no. (%)	17 (70.8)	34 (58.6)	NS (0.299)
ALI, no. (%)	13 (54.2)	15 (25.9)	0.014
MODS, no. (%)	18 (75.0)	37 (63.8)	NS (0.326)
Revised Atlanta classification			NS (0.230)
Mild, no. (%)	0 (0)	0 (0)	
Moderate severe, no. (%)	2 (8.3)	11 (19.0)	
Severe, no. (%)	22 (91.7)	47 (81.0)	
Mortality, no. (%)	13 (54.2)	18 (31.0)	0.049
Hospital duration, days	35 (19.5–83.75)	52 (27–78)	NS (0.343)
ICU duration, days	24.5 (12.75–46.25)	35 (18.25–53.25)	NS (0.375)
Cost, thousand CNY	293 (183–459)	416 (256–553)	NS (0.433)

*PT* prothrombin time, *APTT* activated partial thromboplastin time, *INR* international normalized ratio, *ALT* alanine transaminase, *AST* aspartate transaminase, *BUN* blood urea nitrogen, *APACHE* acute physiology and chronic health evaluation, *IPN* infected pancreatic necrosis, *SVT* splanchnic venous thrombosis, *ACS* abdominal compartment syndrome, *ARDS* acute respiratory distress syndrome, *AKI* acute kidney injury, *ALI* acute liver injury, *MODS* multiple organ dysfunction syndrome, *ICU* intensive care unit. Data are expressed as median (interquartile range) for continuous variables and frequencies (proportions) for categorical variables, as appropriate. Mann–Whitney U-test and Pearson test was used as the circumstances required.
